# Out-of-season increase of puerperal fever with group A *Streptococcus* infection: a case–control study, Netherlands, July to August 2018

**DOI:** 10.2807/1560-7917.ES.2020.25.40.1900589

**Published:** 2020-10-08

**Authors:** Jossy van den Boogaard, Susan JM Hahné, Margreet JM te Wierik, Mirjam J Knol, Sooria Balasegaram, Brechje de Gier

**Affiliations:** 1 National Institute for Public Health and the Environment (RIVM), Bilthoven, Netherlands; 2 European Programme for Intervention Epidemiology Training (EPIET), European Centre for Disease Prevention and Control (ECDC), Stockholm, Sweden; 3 Public Health England, London, United Kingdom

**Keywords:** group A Streptococcus, puerperal fever, impetigo, Netherlands

## Abstract

We observed an increase in notifications of puerperal group A *Streptococcus* (GAS) infections in July and August 2018 throughout the Netherlands without evidence for common sources. General practitioners reported a simultaneous increase in impetigo. We hypothesised that the outbreak of puerperal GAS infections resulted from increased exposure via impetigo in the community.

We conducted a case–control study to assess peripartum exposure to possible, non-invasive GAS infections using an online questionnaire. Confirmed cases were recruited through public health services while probable cases and controls were recruited through social media. We calculated odds ratios (OR) and 95% confidence intervals (95% CI) with logistic regression analysis.

We enrolled 22 confirmed and 23 probable cases, and 2,400 controls. Contact with persons with impetigo were reported by 8% of cases and 2% of controls (OR: 3.26, 95% CI: 0.98–10.88) and contact with possible GAS infections (impetigo, pharyngitis or scarlet fever) by 28% and 9%, respectively (OR: 4.12, 95% CI: 1.95–8.68). In multivariable analysis, contact with possible GAS infections remained an independent risk factor (aOR: 4.28, 95% CI: 2.02–9.09).

We found an increased risk of puerperal fever after community contact with possible non-invasive GAS infections. Further study of this association is warranted.

## Background

Group A *Streptococcus* (GAS, or *Streptococcus pyogenes*) infection can manifest as invasive and non-invasive disease. Oropharynx and skin are the primary colonisation sites from which transmission to a new host occurs, either via respiratory droplets or by skin contact [1,2]. Non-invasive infections with GAS such as impetigo, pharyngitis and scarlet fever are common [1,3,4]. Invasive GAS infections such as streptococcal toxic shock syndrome (STSS), meningitis and fasciitis necroticans are rare but have a high morbidity and mortality. The incidence of invasive GAS infections is estimated to be 2.45 per 100,000 person-years in high-income countries, but seasonal variations and temporary increases of specific *emm* types have been described [3,5-7].

Women in the first 30 days of puerperium have a 20-fold increased risk of invasive GAS infection compared with non-pregnant women of reproductive age [8,9]. Invasive GAS infections in puerperium often present as endometritis or sepsis with a genital focus, but can also manifest at a non-genital site, and as GAS bacteraemia without a clear focus [8,10]. The source of invasive GAS infection in puerperium is often the woman’s throat or that of a close contact [11]. However, healthcare workers carrying GAS are also a possible source, and maternity ward clusters of puerperal GAS infections occur occasionally [9].

European surveillance of puerperal fever with GAS is complicated by differences in notification criteria and definitions used between countries [12,13]; hence, no structural, cross-European surveillance programme for puerperal fever exists and its incidence in Europe is difficult to assess. The European Union-funded Strep-EURO programme that encompassed population-based surveillance of invasive GAS disease in 11 European countries in 2003 and 2004 [14] is no longer active.

In the Netherlands, the notification criteria were changed mid-2016 from puerperal sepsis (sepsis post-partum with GAS cultured from a normally sterile site, or with GAS cultured from a normally non-sterile site without another microorganism that can explain the disease) to all fever within 3 weeks post-partum with GAS cultured from the urogenital tract or from a normally sterile site within 3 weeks of childbirth, to increase the sensitivity of cluster detection [15]. This change resulted in an overall increase in notifications of puerperal GAS infection (ranging from 1–11 notifications/month), with retention of its seasonality (i.e. peak incidences in early spring, decreasing until autumn, to increase again over winter). The incidence of puerperal GAS infections was 32 per 100,000 live births in 2016 and 54 per 100,000 live births in 2017 in the Netherlands (data not shown).

## Outbreak detection

**Figure 1:**
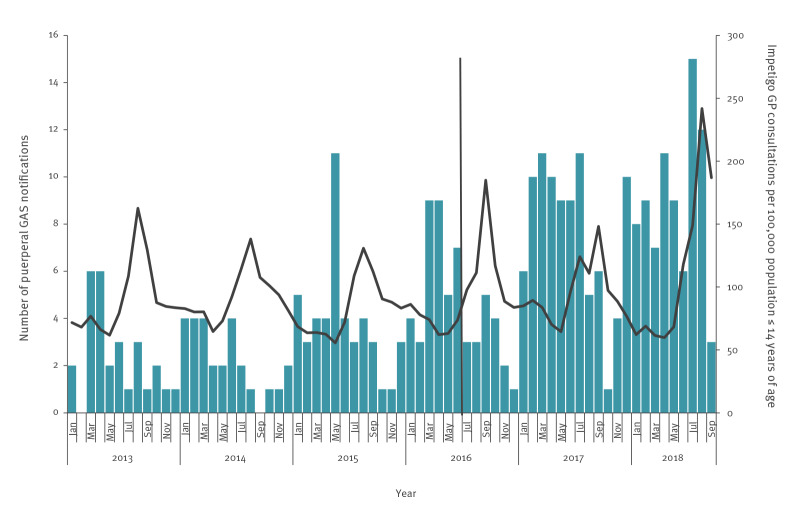
Number of puerperal GAS infection notifications per month and weekly general practitioner consultations rates for impetigo in children ≤ 14 years of age averaged by month, Netherlands, January 2013–September 2018

GAS: Group A *Streptococcus*; GP: general practitioner.

An out-of-season increase in notifications of puerperal GAS infections was observed in July and August 2018, comprising 27 notifications. In the same 2 months in the previous 7 years, there were only 7 cases on average. Even after the change in notification criteria in 2016, the increase in notifications in July and August 2016 (n = 6) and 2017 (n = 16) was not as strong as that seen in July and August 2018 (Figure 1). We contacted neighbouring countries (Belgium, the United Kingdom (UK) and Germany), but no specific increase in puerperal GAS was observed in the summer of 2018 in these countries.

The vertical line indicates the change of puerperal GAS infection notification criteria on 1 July 2016.

Sources: National Institute for Health Services Research (Nivel) Primary Care Database for impetigo and National Institute for Public Health and the Environment (RIVM) OSIRIS (national surveillance database) for puerperal GAS.

The 27 cases occurred throughout the country. Isolates from four cases in the north-east of the Netherlands were typed, revealing three different *emm* types (89.0 in two cases, 87.0 and 75.0). We did not identify common healthcare providers that could have been a source of clusters within the group of 27 cases. A potential hospital cluster of three cases in the south-east was investigated. These cases had the same *emm* type (102.2), but screening of healthcare personnel did not result in the identification of a GAS carrier. The 27 cases had a median age of 31 years (range: 24–39). The median number of days between childbirth and onset of symptoms was 2 (range: −2 to 6). The 27 cases did not differ considerably from the 50 cases in the previous 6 months (January–June 2018) with respect to age, number of days between childbirth and date of onset, or specimen from which GAS was cultured. All 27 cases survived, but one newborn of a mother with puerperal GAS infection died of invasive GAS disease.

There was no simultaneous increase in other notifiable invasive GAS infections (fasciitis necroticans and STSS) in the Netherlands. However, in the same period, general practitioners (GPs) reported a larger number of patients with impetigo (International Classification of Primary Care (ICPC) code S84) than expected for this time of the year (Figure 1) [16]. They did not observe an increase in pharyngitis, scarlet fever, tonsillitis or sore throat (ICPC codes R72.01 and R72.02 for streptococcal pharyngitis or scarlet fever; R74.01 and R74.02 for common cold and acute pharyngitis; and R21.01 for sore throat).

We hypothesised that the surplus of puerperal GAS infections in July and August 2018 had resulted from increased exposure to GAS via impetigo, and conducted a nationwide population-based case–control study to test this hypothesis. Here we describe the results of this case-control study.

## Methods

### Study design and participants

The case–control study was conducted in October and November 2018 using an online questionnaire. Women who gave birth between 1 July 2018 and 31 August 2018 and who were resident in the Netherlands were eligible to participate. Confirmed cases were defined as culture-confirmed cases of puerperal GAS infection notified by laboratories and clinicians via Municipal Public Health Services (MHS) to the National Institute for Public Health and the Environment (RIVM), fulfilling the following notification criteria: fever within 21 days post-partum and isolation of GAS from a normally sterile body site or urogenital tract [15]. Probable cases were women who self-reported to have had puerperal fever for which they received antibiotics within 21 days post-partum, and who were identified through the online questionnaire. Controls were defined as women without fever within 21 days after delivery. Cases were asked to participate by the MHS; controls were recruited through Facebook and Twitter accounts of the RIVM. We aimed to enrol all cases (n = 27) and at least four times as many controls for sufficient statistical power.

Cases and controls filled in an identical questionnaire, which included questions about medical history, recent pregnancy and delivery, and possible exposure to GAS through contact with children or adults with impetigo, pharyngitis or scarlet fever between 1 week before and 1 week after delivery.

### Ethical statement

The study protocol was submitted to the Centre for Clinical Expertise at RIVM and exempted from further approval by the ethical research committee according to the Dutch law for medical research involving human subjects.

### Data analysis

Univariable and multivariable logistic regression analyses were used to assess the odds ratios (OR) and 95% confidence intervals (95% CI) of different exposures among cases of puerperal GAS infection compared with controls. The population-attributable fraction was estimated by using the proportion exposed among controls as a proxy for the proportion of the population exposed [17]. The primary exposure variables of interest were contacts with persons with impetigo or other possible non-invasive GAS infections (pharyngitis, scarlet fever) between 1 week before and 1 week after delivery. Whenever the participant replied ‘I don’t know’, the answers were set to ‘missing’ in the data analysis. Other exposure variables considered were place of delivery, type of delivery, type of birth attendant, bathing during labour or childbirth, manual placenta removal, intrapartum antibiotics, hospitalisation following delivery, and type and number of healthcare providers until 7 days after delivery.

We performed multivariable logistic regression analysis to explore possible confounding by or interaction with various demographic characteristics. We undertook sensitivity analyses by repeating the analyses with confirmed cases only and also by recoding ‘I don’t know’ to ‘no’. Analyses were done in Stata version 15.1 (StataCorp, Texas, United States (US)).

## Results

**Figure 2:**
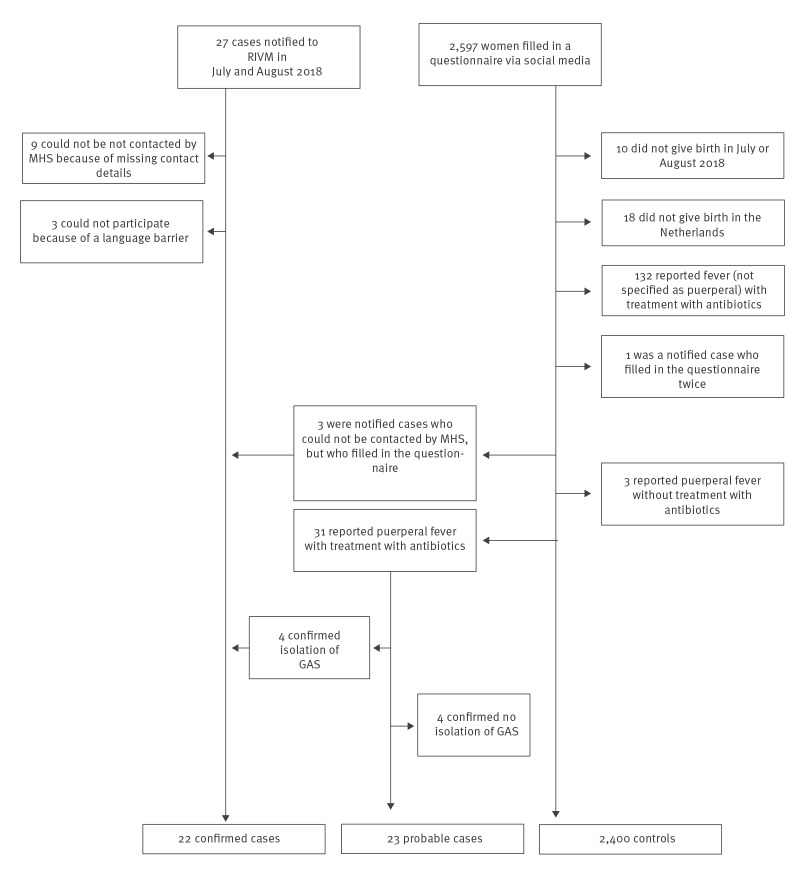
Flow diagram of recruitment of cases and controls for GAS infection case–control study, Netherlands, July–August 2018

GAS: group A *Streptococcus*; MPS: Municipal Public Health Services; RIVM: National Institute for Public Health and the Environment.

Of 27 notified cases, 18 completed the questionnaire. Twelve could not be reached because of a language barrier (n = 3) or because of missing contact details (n = 9). However, three of the nine with missing contact details filled in the questionnaire for controls distributed via social media on their own initiative, leaving a total of nine notified cases who were not reached. For the recruitment of controls, the link to the online questionnaire went live on social media on 18 October 2018. At closure on 25 October, 2,597 women had filled in the questionnaire, of whom 167 were excluded because they did not fulfil the inclusion criteria (Figure 2).

Unexpectedly, 31 women self-reported puerperal fever for which they received oral or intravenous antibiotics within 21 days after delivery. Of these, 18 replied to our additional questions about who diagnosed the puerperal fever and whether it was laboratory confirmed. Four women self-reported that GAS was cultured and were thus included as confirmed cases while four indicated that no GAS was grown on culture were thus excluded from the study. The other 10 did not know whether laboratory tests were done or what the results were. They and the 13 women who did not respond to the additional questionnaire remained probable cases (Figure 2).

**Figure 3:**
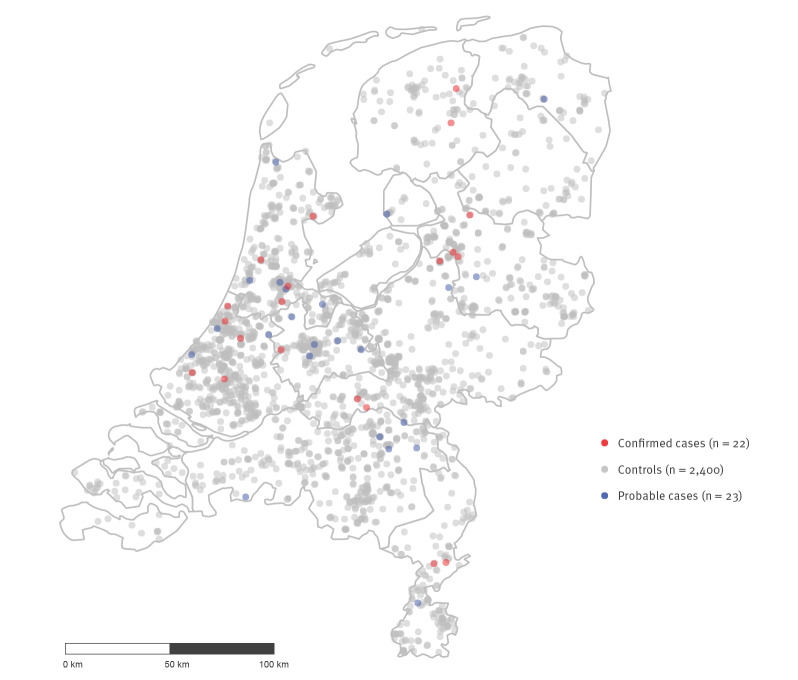
Municipality of residence of confirmed (n = 22) and probable (n = 23) GAS infection cases and controls (n = 2,400) based on four-digit postal codes, Netherlands, July–August 2018

Hence, 22 confirmed cases, 23 probable cases and 2,400 controls were included for analysis (Figure 2). Cases and controls resided across the Netherlands (Figure 3). They neither differed significantly in demographic variables (Table 1) nor in the healthcare setting where they gave birth nor in the type of delivery (Table 2). Contact with a person with impetigo was reported by 3 (8%) cases (probable and confirmed) and 57 (2%) controls (OR: 3.26, 95% CI: 0.98–10.88); contact with a person with pharyngitis by 6 (16%) cases and 125 (6%) controls (OR: 3.02, 95% CI: 1.24–7.36); and contact with a child with scarlet fever by 1 (2%) case and 2 (0.1%) controls (OR: 27.70, 95% CI: 2.46–311.14) (Table 2). In total, 28% of cases and 9% of controls reported a contact with a possible GAS infection (OR: 4.12, 95% CI: 1.95–8.68) (Table 2). The estimated proportion of puerperal fever cases attributable to contact with a possible GAS contact (impetigo, pharyngitis or scarlet fever) was 22% (calculated by using the formula for population attributable percent in case-control studies [18]). Contacts with impetigo were mostly children for both cases (3/3) and controls (39/57), and contacts with pharyngitis were more often adults than children (4/6 cases reported adult contact while controls reported 58/125 adult contacts, 47/125 child contacts and 20/125 adult and child contacts) (Supplementary Table S1).

GAS: group A *Streptococcus*.

**Table 1:** Characteristics of participants in the GAS infection case–control study, Netherlands, July–August 2018 (22 confirmed cases, 23 probable cases, 2,400 controls)

Characteristics		Confirmed cases (n = 22)		Probable cases (n = 23)		Controls (n = 2,400)		p value^a^
		Median or n	IQR or %	Median or n	IQR or %	Median or n	IQR or %	
Median age (years)		31	29–34	31	26–36	31	28–33	0.87
Median number of household members^b^		4	3–4	4	3–5	4	3–4	0.40
Median number of children in household^c^		2	1–2	2	1–3	2	1–2	0.43
Two or more children in household^c^		15	68	12	52	1,335	56	0.47
Born abroad		1	5	0	0	72	3	0.85
Educational level^d^	Low	2	9	1	4	140	6	0.87
	Medium	8	36	11	48	836	35	
	High	12	55	11	48	1,400	58	
	Unknown	0	0	0	0	24	1	

GAS: group A *Streptococcus*; IQR: interquartile range.

^a^ Mann Whitney U test (comparison of medians) and Chi square test for comparison of proportions.

^b^ Including the woman herself and the newborn.

^c^ Including the newborn.

^d^ Low: no education, primary education, junior technical school or lower general vocational secondary education; medium: intermediate vocational secondary education, higher vocational or higher general secondary education; high: pre-university or university education.

**Table 2:** Univariable analysis of possible risk factors of puerperal GAS infection, confirmed and probable cases compared with controls, Netherlands, July–August 2018 (45 confirmed and probable cases; 2,400 controls)

Exposure variable	Confirmed and probable cases (n = 45)			Controls (n = 2,400)			Univariable analysis	
	Exposed	Total^a^	%	Exposed	Total^a^	%	OR	95% CI
**Possible exposure to GAS**								
Contact with child/adult with impetigo	3	40	8	57	2,349	2	3.26	0.98–10.88
Contact with child/adult with pharyngitis	6	37	16	125	2,073	6	3.02	1.24–7.36
Contact with child with scarlet fever	1	44	2	2	2,383	0.1	27.70	2.46–311.14
Any contact with child/adult with possible GAS infection^b^	10	36	28	176	2,061	9	4.12	1.95–8.68
Woman had impetigo herself	1	45	2	11	2,393	0.5	4.92	0.62–38.95
Woman had pharyngitis herself	1	44	2	105	2,354	4	0.50	0.07–3.65
Any contact with someone with varicella	1	43	2	41	2,357	2	1.34	0.18–10.01
**Factors related to pregnancy and delivery**								
Twin pregnancy	1	45	2	30	2,400	1	1.80	0.24–13.46
Prematurity	0	41	0	94	2,332	4	NA	NA
Healthcare provider in week before delivery: midwife	38	45	84	1,901	2,400	79	1.42	0.63–3.21
Healthcare provider in week before delivery: gynaecologist	13	45	29	866	2,400	36	0.72	0.38–1.38
Gave birth at home	10	45	22	478	2,400	20	1.15	0.56–2.33
Gave birth in birth centre (primary care)	14	45	31	781	2,400	33	0.94	0.50–1.77
Gave birth in hospital (secondary care)	21	45	47	1,140	2,400	48	0.97	0.54–1.75
Bathing during labour (at any place)	11	44	25	497	2,368	21	1.25	0.63–2.50
Bathing during labour in hospital	3	44	7	166	2,368	7	0.97	0.30–3.17
Baby born in bath	0	11	0	65	480	14	NA	NA
Artificial ROM	13	45	29	600	2,400	25	1.22	0.64–2.34
ROM > 12 hours	6	45	13	351	2,373	15	0.89	0.37–2.11
Induction of labour	12	45	27	542	2,400	23	1.25	0.64–2.43
Vaginal delivery, spontaneous	38	45	84	1,947	2,400	81	1.26	0.56–2.85
Vaginal delivery, artificial	3	45	7	174	2,400	7	0.91	0.28–2.98
Caesarean section	4	45	9	279	2,400	12	0.74	0.26–2.08
Duration of delivery ≥ 12 hours	15	45	33	736	2,400	31	1.13	0.60–2.11
Perineum rupture	16	45	36	830	2,392	35	1.04	0.56–1.92
Episiotomy	7	45	16	379	2,392	16	0.98	0.43–2.21
Perineum rupture and episiotomy	2	45	4	65	2,392	3	1.67	0.39–7.02
Perineum sutures	24	45	53	1,268	2,400	53	1.00	0.56–1.79
Artificial placenta delivery	0	41	0	77	2,119	4	NA	NA
Preventive antibiotics	2	45	4	117	2,293	5	0.87	0.21–3.61
Hospital admission directly following delivery	23	45	51	1,036	2,400	43	1.38	0.76–2.48
Two or more women in same room during hospitalisation	1	23	4	47	1,036	5	0.96	0.13–7.25
Two or more healthcare providers performing vaginal/perineal care^c^	39	45	87	1,717	2,400	72	2.59	1.09–6.14
Vaginal/perineal care^c^ performed by midwife	40	45	89	2,061	2,400	86	1.32	0.52–3.36
Vaginal/perineal care^c^ performed by hospital staff	35	45	78	1,244	2,400	52	3.25	1.60–6.60
Vaginal/perineal care^c^ performed by healthcare staff at home	7	45	16	479	2,400	20	0.74	0.33–1.66

CI: confidence interval; GAS: group A *Streptococcus*; MPS: Municipal Public Health Services; NA: not applicable; OR: odds ratio; RIVM: National Institute for Public Health and the Environment; ROM: rupture of membranes.

^a^ Excluding women who answered ‘don’t know’ to this question.

^b^ Possible GAS infection: impetigo, pharyngitis and/or scarlet fever.

^c^ Vaginal/perineal care was defined as vaginal examination and/or care of perineal wounds or ruptures between 1 week before and 1 week after delivery.

Vaginal/perineal care (including vaginal examination and care of the perineum between 1 week before and 1 week after delivery) by hospital staff (i.e. gynaecologists, residents in gynaecology, interns and nurses) was more often reported by cases than controls (OR: 3.25, 95% CI: 1.60–6.60). Cases also received vaginal/perineal care by two or more healthcare providers more often than controls (OR: 2.59, 95% CI: 1.09–6.14) (Table 2).

For multivariable analysis, the three variables that were significant in the univariable analysis and of which numbers were large enough (possible GAS contacts, vaginal/perineal care performed by hospital staff, and two or more healthcare providers performing vaginal/perineal care) were included. Of these three variables, only possible GAS contacts (aOR 4.32, 95% CI: 2.04–9.17) and hospital staff providing vaginal/perineal care (aOR 4.62, 95% CI: 1.91–11.17) were independent risk factors. No interactions with or confounding by demographic characteristics or other variables were observed. In sensitivity analysis, trends were similar (Supplementary Tables S2 and S3).

## Outbreak control measures

No immediate outbreak control measures were taken at the time, but taking these findings into account, advice on avoiding contacts with possible GAS infection in puerperium will be reinforced within a revision of the Dutch guidelines on public health management of invasive GAS infections. As puerperal fever notifications were remarkably high again in early 2019, the RIVM requested all puerperal GAS isolates in the Netherlands to be *emm* typed for a pilot period of 2 years to aid cluster detection and the investigation of possible sources.

## Discussion

In our study, women who developed puerperal GAS infection had more contact with a person with a possible non-invasive GAS infection (impetigo, pharyngitis or scarlet fever) than controls. The proportion of puerperal GAS infections attributable to possible GAS contacts was 22%; thus, even though based on small numbers, at least part of the out-of-season increase in puerperal GAS infections in July and August 2018 in the Netherlands could possibly be explained by the usually large number of children with impetigo in this period.

Close contact with persons with a possible (non-invasive) GAS infection has previously been described as a risk factor for puerperal sepsis. Close household contacts probably contribute most to transmission [9,19,20]. One-third of impetigo contacts and all pharyngitis and scarlet fever contacts were household contacts in our study.

Both GAS pharyngitis and impetigo show a seasonal pattern in countries with a moderate climate; pharyngitis predominating in late winter and impetigo in summer [4,21,22]. July and August 2018 were remarkably warm in the Netherlands according to data from the Royal the Netherlands Meteorological Institute (KNMI). Especially in the second half of July, day temperatures reached 35 °C and above [23]. The GP-based surveillance system at the Netherlands Institute for Health Services Research (Nivel) showed a more than average increase in impetigo in this period in all ages and regions [16]. No increase in GP consultations for pharyngitis or scarlet fever were observed in July and August 2018, but we did find exposure to these to be risk factors for developing puerperal fever. Increases in scarlet fever incidences were reported from the UK and several Asian countries, including China, in the last 10 years [24-26]. In the Netherlands, scarlet fever is mostly a clinical diagnosis that is not notifiable. Hence, the true incidence of scarlet fever in the Netherlands is not known.

Vaginal/perineal care by hospital staff as compared to non-hospital staff (including midwives) was reported more often by cases than controls in our study. We are unsure whether this points towards nosocomial transmission because vaginal/perineal care was defined as having taken place between 1 week before and 1 week after delivery. Since the onset of puerperal fever of notified cases occurred with a median of 2 days after delivery, it is likely that some vaginal/perineal care occurred in hospital after disease onset and that it was a consequence rather than a cause of puerperal GAS infection.

The strengths of this study were the quick recruitment of cases and controls with this occurring within 2 months after the outbreak. This would have likely limited the risk of recall bias, and the large number of participating controls. At survey closure, which was 1 week after posting the link to the questionnaire on social media, 2,597 women had responded. This corresponds to almost 9% of all women who gave birth in July and August 2018 in the Netherlands [27]. Social media was shown to be a very effective platform to reach new mothers. The Facebook and Twitter messages were viewed, liked, replied to and/or shared almost 1,800,000 times, and vivid online discussions developed on potential causes of the outbreak.

A limitation of this study was the small number and non-response of confirmed cases, despite efforts to recruit via local teams. We were able to increase power by combining confirmed and probable cases. However, probable cases lacked documented laboratory confirmation. Nevertheless, we know from microbiologists that puerperal GAS infections are not always notified by the laboratory, mainly because of misunderstanding of the notification criteria. One of the four women who self-reported that GAS was cultured, named the hospital and the date of delivery. The hospital confirmed that a culture-confirmed case of puerperal GAS infection in that period had not been notified. Another study limitation was the lack of laboratory confirmation of possible GAS contacts. Impetigo is usually diagnosed on clinical symptoms without laboratory confirmation. Hence, information on the causative pathogens was not available and the relative contributions of GAS and *Staphylococcus aureus* to the increase in impetigo are unknown. In addition, the extent of contact that the women reported to have had with others with possible GAS infections than their household members was not assessed in this study. The risk of GAS transmission is probably smaller through these contacts than through household members. Also, there might have been differential recall bias in this study, despite the quick onset after outbreak detection. It is possible that women whose puerperium was complicated by GAS infection have a differential memory on whether they had been in contact with persons with possible GAS infections compared with controls. Finally, since controls were recruited through social media only, this might have resulted in a selection bias. However, general characteristics of our controls did not differ significantly from those of cases, and as all cases also filled the questionnaire online any potential bias would likely be non-differential between cases and controls.

## Conclusion

In conclusion, our study suggests that contact with non-invasive GAS infections in the community in late pregnancy or puerperium increased the risk of puerperal GAS infection. For confirmation of our findings, future studies should include a larger number of cases and laboratory confirmation of possible GAS contacts, i.e. cultures of impetiginous lesions and throat swabs, as well as typing of GAS isolates found in both puerperal fever cases and contacts. The extent to which nosocomial transmission contributes to puerperal GAS infections should be studied in more detail. We suggest that women in late pregnancy and puerperium avoid physical contact with household members with symptoms of GAS infection as much as possible, and that these household members promptly seek healthcare to have antibiotic treatment initiated, if indicated, in order to prevent puerperal GAS infection.

## Supplementary Data

570000 Supplement
