# Supplementary material for: Out-of-season increase of puerperal fever with group A Streptococcus infection: a case–control study, Netherlands, July to August 2018
Source: Euro Surveill. 2020 Oct 8;25(40):1900589. doi: 10.2807/1560-7917.ES.2020.25.40.1900589 (PMC7545820; doi:10.2807/1560-7917.ES.2020.25.40.1900589)
Supplement: Supplement [file 1900589-Supp1.pdf]

This supplementary material is hosted by *Eurosurveillance* as supporting information alongside the article "Out of season increase of puerperal fever with group A Streptococcus in July-August 2018 in the Netherlands: a case-control study" on behalf of the authors who remain responsible for the accuracy and appropriateness of the content. The same standards for ethics, copyright, attributions and permissions as for the article apply. *Eurosurveillance* is not responsible for the maintenance of any links or email addresses provided therein.

Supplementary table 1. Type of contact (child or adult) with a potential GAS infection of cases (confirmed and probable cases) and controls

		Impetigo		Sore throat		Scarlet fever	
		**Cases**	**Controls**	**Cases**	**Controls**	**Cases**	**Controls**
Total exposed		3	57	6	125	1	2
Type of contact:	Child	3	39	2	47	1	2
	Adult	0	15	4	58	0	0
	Child and adult	0	3	0	20	0	0
GAS contact with > 1 child		0	8	0	7	0	0
Child in own household		1	21	2	52	1	0
Child of family member		1	18	0	12	0	0
Child in neighbourhood		1	9	0	8	0	2

Supplementary table 2. Univariable analysis of possible risk factors of puerperal GAS infection, confirmed cases compared to controls

**Exposure**	**Confirmed cases (n=22)**	**Controls (n=2,400)**	**Univariable analysis**
**Variable**	**Exposed / Total[1] (%)**	**Exposed / Total[1] (%)**	**OR (95% CI)**
Possible exposure to GAS			
Contact with child/adult with impetigo	1/17 (6%)	57/2,349 (2%)	2.51 (0.33-19.3)
Contact with child/adult with pharyngitis	2/19 (11%)	125/2,073 (6%)	1.83 (0.42-8.02)
Contact with child with scarlet fever)	1/22 (5%)	2/2,383 (0.1%)	56.69 (4.95-649.50)

Any contact with child/adult with possible GAS infection[2]	4/17 (24%)	176/2,061 (9%)	3.30 (1.06-10.21)
Woman had impetigo herself	1/22 (5%)	11/2,393 (0.5%)	10.31 (1.27-83.51)
Woman had pharyngitis herself	1/21 (5%)	105/2,354 (4%)	1.07 (0.14-8.06)
Any contact with someone with varicella	0/20 (0%)	41/2,357 (2%)	-
Factors related to pregnancy and delivery			
Twin pregnancy	0/22 (0%)	30/2,400 (1%)	-
Prematurity	0/22 (0%)	94/2,332 (4%)	-
Healthcare provider in week before delivery: midwife	21/22 (95%)	1,901/2,400 (79%)	5.51 (0.74-41.08)
Healthcare provider in week before delivery: gynaecologist	4/22 (18%)	866/2,400 (36%)	0.39 (0.13-1.17)
Gave birth at home	6/22 (27%)	478/2,400 (20%)	1.51 (0.59-3.87)
Gave birth in birth centre (primary care)	8/22 (36%)	781/2,400 (33%)	1.18 (0.49-2.84)
Gave birth in hospital (secondary care)	8/22 (36%)	1,140/2,400 (48%)	0.63 (0.26-1.51)
Bathing during labour	5/22 (23%)	497/2,368 (21%)	1.11 (0.41-3.01)
Bathing during labour in hospital	1/22 (5%)	166/2,368 (7%)	0.63 (0.08-4.73)
Baby born in bath	0/5 (0%)	65/480 (14%)	-
Artificial ROM	8/22 (36%)	600/2,400 (25%)	1.71 (0.72-4.11)
ROM > 12 hours	3/22 (14%)	351/2,373 (15%)	0.91 (0.27-3.09)
Induction of labour	5/22 (23%)	542/2,400 (23%)	1.01 (0.37-2.75)
Vaginal delivery, spontaneous	20/22 (91%)	1,947/2,400 (81%)	2.33 (0.54-9.99)
Vaginal delivery, artificial	1/22 (5%)	174/2,400 (7%)	0.61 (0.08-4.56)
Caesarean section	1/22 (5%)	279/2,400 (12%)	0.36 (0.05-2.70)
Duration of delivery ≥12 hours	7/22 (32%)	736/2,400 (31%)	1.06 (0.43-2.60)
Perineum rupture	7/22 (32%)	830/2,392 (35%)	0.88 (0.36-2.16)
Episiotomy	4/22 (18%)	379/2,392 (16%)	1.18 (0.40-3.51)
Perineum rupture and episiotomy	1/22 (5%)	65/2,392 (3%)	1.70 (0.23-12.87)
Perineum sutures	12/22 (55%)	1,268/2,400 (53%)	1.05 (0.46-2.40)
Artificial placenta delivery	0/21 (0%)	77/2,119 (4%)	-
Preventive antibiotics	1/22 (5%)	117/2,293 (5%)	0.89 (0.12-6.64)
Hospital admission directly following delivery	9/22 (41%)	1,036/2,400 (43%)	0.91 (0.39-2.14)

≥2 women in same room during hospitalisation	1/9 (11%)	47/1,036 (5%)	2.63 (0.32-21.47)
≥2 healthcare providers performing vaginal/perineal care[3]	19/22 (86%)	1,717/2,400 (72%)	2.52 (0.74-8.54)
Vaginal/perineal care[3] performed by midwife	21/22 (95%)	2,061/2,400 (86%)	3.45 (0.46-25.76)
Vaginal/perineal care[3] performed by hospital staff	17/22 (77%)	1,244/2,400 (52%)	3.16 (1.16-8.59)
Vaginal/perineal care[3] performed by healthcare staff at home	4/22 (18%)	479/2,400 (20%)	0.89 (0.30-2.65)

[1] Excluding women who answered "don't know" to this question

[2] Possible GAS infection = impetigo, pharyngitis and/or scarlet fever

[3] Vaginal/perineal care was defined as vaginal examination and/or care of perineal wounds or ruptures between one week before and one week after delivery

ROM = Rupture of Membranes; OR = odds ratio ; 95% CI = 95% confidence interval

Supplementary table 3. Univariable analysis of possible risk factors of puerperal GAS infection, cases (confirmed cases and probable cases) compared to controls, when answers "unknown" set to "no"

**Exposure**	**Cases** (n=45)	**Controls** (n=2,400)	**Univariable analysis**
**Variable**	**Exposed / Total[1] (%)**	**Exposed / Total[1] (%)**	**OR (95% CI)**
Possible exposure to GAS			
Contact with child/adult with impetigo	3/45 (7%)	57/2,400 (2%)	2.94 (0.88-9.75)
Contact with child/adult with pharyngitis	6/45 (13%)	125/2,400 (5%)	2.80 (1.16-6.74)
Contact with child with scarlet fever)	1/45 (2%)	2/2,400 (<0.01%)	27.25 (2.43-306.12)
Any contact with child/adult with possible GAS infection[2]	10/45 (22%)	176/2,400 (7%)	3.61 (1.76-7.41)
Woman had impetigo herself	1/45 (2%)	11/2,400 (0.5%)	4.94 (0.62-39.07)
Woman had pharyngitis herself	1/45 (2%)	105/2,400 (4%)	0.50 (0.068-3.64)
Any contact with someone with varicella	1/45 (2%)	41/2,400 (2%)	1.31 (0.18-9.72)

[1] The answers "don't know" set to "no"

[2] Possible GAS infection = impetigo, pharyngitis and/or scarlet fever

OR = odds ratio ; 95% CI = 95% confidence interval
